# Redescription and Molecular Characterisation of *Derogenes ruber* Lühe, 1900 (Hemiuroidea: Derogenidae) from *Chelidonichthys lastoviza* (Scorpaeniformes: Triglidae) in the Western Mediterranean

**DOI:** 10.1007/s11686-023-00749-z

**Published:** 2023-12-08

**Authors:** Kamilia Gharbi, Chahinez Bouguerche, Mohammed Ahmed, Gerardo Pérez-Ponce de León, Fadila Tazerouti

**Affiliations:** 1 Laboratoire de Biodiversité et Environnement: Interactions-Génomes, Faculté des Sciences Biologiques, Université des Sciences et de la Technologie Houari Boumediene, BP 32, El Alia Bab Ezzouar, Alger, Algérie; 2 Department of Zoology, Swedish Museum of Natural History, Stockholm, Sweden; 3 Departamento de Zoología, Instituto de Biología, Universidad Nacional Autonoma de México, Ciudad de México, Mexico; 4 Escuela Nacional de Estudios Superiores Unidad Mérida, Universidad Nacional Autonoma de México, Ucú, Mexico; 5 Institut Systématique Évolution Biodiversité (ISYEB), Muséum National d’Histoire Naturelle, 57 rue Cuvier, CP 51, 75005 Paris, France; 6 Department of Evolution, Ecology and Behaviour, Institute of Infection, Veterinary and Ecological Sciences, University of Liverpool, Liverpool, L69 7AB UK

**Keywords:** Derogenidae, Mediterranean, *Derogenes ruber*, *Derogenes latus*, Morphology, 28S rDNA, ITS2, *Cox*1

## Abstract

**Purpose:**

*Derogenes ruber* Lühe, 1900, the type-species of the genus *Derogenes* Lühe, 1900, is a poorly known derogenid digenean. The original description of this species was not illustrated and aspects of the morphology of the parasite from the type-host remain scarce. Available records of this species were brief and/or lacked illustrations and were based on morphology alone. Additionally, molecular data for *Derogenes* spp. are warranted to untangle species complexes as they provide a better assessment of interspecific genetic divergence.

**Methods:**

*Derogenes ruber* is redescribed based on newly collected specimens from the gall bladder of its type-host *Chelidonichthys lastoviza* (Bonnaterre, 1788) collected in the Western Mediterranean off the Algerian coast during 2017–2019 and molecular data are provided using a partial fragment of the nuclear 28S ribosomal RNA gene (28S rRNA), the internal transcribed spacer 2 (ITS2) and a fragment of the mitochondrial cytochrome *c* oxidase subunit 1 (*cox*1) gene.

**Results:**

We herein provide a detailed illustrated redescription and morphometric data of *D. ruber* from its type-host *C. lastoviza*. We report a new geographical record (off Algeria) for it. *Derogenes ruber* is also genetically characterised for the first time. Species/lineages of *Derogenes* were recovered in five strongly supported reciprocally monophyletic clades: (i) *D. ruber* from *C. lastoviza* off Algeria; (ii) *D. lacustris* from *Galaxias maculatus* (Jenyns) off Argentina; (iii) Lineage “*D. varicus* DV1” (*D. varicus *sensu stricto) from fish hosts in the White and Barents seas and the North Sea; (iv) Lineage “*D. varicus* DV2” from mollusc hosts in the White Sea; and (v) Lineage “*D. varicus* DV3” from *Eumicrotremus fedorovi* Mandrytsa. in the Pacific Ocean. Hence, comparison of the newly generated sequences with other available data for *Derogenes* species supports the distinction of *D. ruber* confirming its taxonomic status and helping assess interspecific variation. Comparison of *D. ruber* with the closely related species *Derogenes latus* revealed overlaps in morphometric data and the validity of the latter species is questioned.

**Conclusion:**

The combination of morphological and molecular data provided for *D. ruber* provides a firm foundation for further investigations of *Derogenes* spp. Although we do describe herein material of *D. ruber* from the type-host, given that the occurrence of a single *Derogenes* species in various hosts has been challenged by molecular data, and both *D. lacustris* and *D. varicus *sensu stricto had been genetically proven to occur in various hosts, *D. ruber* and *D. latus* may be indeed synonymous. Additional sequencing effort on *Derogenes* spp. will strengthen systematic comparative studies and evolutionary relationships within the Derogenidae in general.

## Introduction

Derogenids are hemiuroid digenean gut parasites, occurring in fishes. Throughout most of their taxonomic history, they were accommodated within a broad concept of the family Hemiuridae Looss, 1899 [1]. The Derogenidae Nicoll, 1910 was first used at full family rank by Dollfus [2] but was initially erected at the subfamily level by Nicoll [3] as the Derogeninae Nicoll, 1910 (referred to as the Derogeninae Dollfus, 1950 by Skrjabin and Guschanskaja [4]). The latter authors included the subfamily within the Halipegidae Poche, 1926, but the Derogenidae has priority [1].

Gibson and Bray [5] established the initial foundational classification of the Derogenidae, offering identification keys for its subfamilies and genera. Within this context, Gibson [1] acknowledged the presence of three subfamilies: Derogeninae Nicoll, 1910, Halipeginae Poche, 1926, and Gonocercinae Skrjabin & Guschanskaja, 1955. At present, the Derogenidae comprises only two subfamilies: Halipeginae Poche, 1926, and Derogeninae [1]. This adjustment in classification occurred due to a molecular study conducted by Sokolov *et al.* [6], who elevated the Gonocercinae to the status of a full family.

Five valid genera are included in the Derogeninae: *Derogenes* Lühe, 1900, *Gonocercella* Manter, 1940 [7], *Leurodera* Linton, 1910, *Progonus* Looss, 1899, and *Derogenoides* Nicoll, 1913 [1, 8].

Records of derogenine derogenids in the Mediterranean are rare [9]. Thus, previous records of *Derogenes* spp. in this region include *D. adriaticus* Nikolaeva, 1966, *D. crassus* Manter, 1934, *D. fuhrmanni* Mola, 1912, *D. latus* Janiszewska, 1953, *D. minor* Looss, 1901, *D. ruber* Lühe, 1900, and *D. varicus* (Müller, 1784) [10, 11]. However, most of the records lack morphological information justifying correct species identification and making the assessment of host–parasite associations difficult if not impossible. For example, only in the Mediterranean, *D. varicus* has been reported in 15 hosts of 13 unrelated fish families [11] indicating that this “generalist” species may represent a species complex. This has been suggested by Bray *et al.* [12] and Køie [13], and a recent study based on multigene sequence data supported this suggestion by providing evidence for the existence of four genetic lineages of *D. varicus* [14, 15].

*Derogenes ruber*, the type-species of the genus was less frequently encountered and reported. The type-material of *D. ruber* was described by Lühe [16] from the gall-bladder of the streaked gurnard *Trigla lineata* Gmelin, 1789 (a junior synonym of *Chelidonichthys lastoviza* (Bonnaterre, 1788)) off Rovinj, Croatia, Adriatic Sea. This trematode is known from the short original description that lacked illustrations, and a subsequent general illustration, based on a record and identification from a different host, the piper gurnard *Trigla lyra* L. from a close locality, off Split, Croatia [17]. Bouguerche *et al.* [15] redescribed this species based only on two specimens found in Arthur Looss’s collection and did not thus provide any molecular data. Other reports of this derogenid are from the North-East Atlantic (off Azores, Canary and Cape Verde Islands [18], and off Spain [19].

During parasitological surveys of helminths of fishes from off the southern coasts of the Western Mediterranean off Algeria, we collected representatives of *D. ruber* from the gall bladder of its type-host, *C. lastoviza*. The aim of the present study is to provide a formal redescription of *D. ruber* and to characterise the species genetically based on partial 28S ribosomal RNA gene (28S rRNA), internal transcribed spacer ITS2, and a fragment of the mitochondrial cytochrome *c* oxidase subunit 1 (*cox*1) gene sequences.

## Materials and Methods

### Collection and Sampling of Fish

A total of 168 specimens of *C. lastoviza* were collected during 2017–2019, from local fishermen immediately after capture in different regions off the Algerian coast: Ghazaouet (35° 06′ 0′′ N, 1° 51′ 0′′ W), Cherchell (36° 36′ 31′′ N, 2° 11′ 50′′ E), Bouharoune (36° 37′ 24′′ N, 2° 39′ 17′′ E), Alger (36° 45′ 8′′ N, 3° 2′ 31′′ E), Bordj el Bahri (36° 47′ 26′′ N, 3° 14′ 59′′ E), Ain Taya (36° 47′ 30′′ N, 3° 17′ 20′′ E), Reghaia (36° 43′ 60′′ N, 3° 21′ 0′′ E), Cap Djinet (36° 52′ 37′′ N, 3° 43′ 23′′ E), and Dellys (36° 54′ 48′′ N, 3° 54′ 51′′ E). Fish specimens were kept on ice and transferred immediately to the laboratory, identified using the key [20, 21], and examined on the day of purchase. Viscera were placed in separate Petri dishes containing seawater and observed under a Zeiss microscope for the presence of digeneans.

### Morphological Methods

Live digeneans were killed and fixed in near-boiling water. Specimens for morphological analysis were fixed under cover-glass pressure in Bouin’s fluid [10], then preserved in 70% ethanol, stained with acetic carmine, dehydrated through a graded alcohol series, cleared in clove oil, and mounted in Canada balsam as permanent mounts. Five specimens were preserved immediately in 96% ethanol for molecular characterisation and were processed as hologenophores (sensu Pleijel *et al.* [22]).

Permanent mounts of the hologenophores, consisting of 2/3 of the body (posterior third excised and used for sequencing), stained and mounted in Canada balsam. Drawings were made using a Zeiss microscope (Université des Sciences et de la Technologie Houari Boumediene, USTHB) and a Nikon Eclipse i80 microscope with DIC (differential interference contrast) (Swedish Museum of Natural History, SMNH) equipped with a drawing tube, and scanned and redrawn with Adobe Illustrator 2023, version 28.0.

Measurements are in given in micrometres and presented as the range followed by the mean in parentheses. Voucher material was deposited at the Swedish Museum of Natural History (SMNH), Stockholm, Sweden under accession numbers SMNH 218781–SMNH 218 805.

### Molecular Methods

Genomic DNA was extracted from a total of five hologenophores, and genetic sequence data were generated for three genetic markers: a partial region of the mitochondrial cytochrome *c* oxidase subunit 1 gene (*cox*1), the second internal transcribed spacer region (ITS2 rDNA), and the large (28S) ribosomal RNA gene. A small fragment of each hologenophore (posterior third) was placed in a 1.5 ml microcentrifuge tube containing 20 μL buffer ATL (Qiagen, Hilden, Germany). For extraction of genomic DNA (gDNA), 20 μL buffer ATL and 20 μL proteinase K were added to each sample, followed by vortexing and incubation in an incubating microplate shaker at 56 °C and 300 rpm overnight. The lysed samples were processed to obtain gDNA following the manufacturer’s instructions for gDNA extraction using the Qiagen QiAmp DNA Microkit. Polymerase chain reaction (PCR) amplification was performed in 25 µl reaction mix using Illustra Hot Start Mix RTG (0.2 µl) reaction kit (GE Healthcare Life Sciences, Uppsala, Sweden). The reaction mix consisted of 1 µl (0.4 µM) of each primer, 2 µl template DNA, and 21 µl nuclease-free water. The primer set JB3 (5′-TTT TTT GGG CAT CCT GAG GTT TAT-3′) and COI R-Trema (5′-CAA CAA ATC ATG ATG CAA AAG G-3′) were used to amplify a fragment the *cox*1 gene [23]. The thermocycling profile consisted of an initial denaturation step at 94 °C for 5 min, followed by 35 cycles of denaturation at 94 °C for 30 s, annealing at 45 °C for 30 s, and extension at 72 °C for 1 min, with a final extension step at 72 °C for 10 min [14]. Primers, amplification, and sequencing protocols for the 28S rDNA region followed Pérez-Ponce de León *et al.* [24] and García-Varela and Nadler (2005) [25]. The thermocycling profile consisted of an initial denaturation step at 94 °C for 3 min, followed by 35 cycles of denaturation at 94 °C for 60 s, annealing at 54 °C for 60 s, and extension at 72 °C for 1 min, with a final extension step at 72 °C for 7 min. ITS2 rDNA spacer was amplified using the primers 3S [26] and ITS2.2 [27] and the following thermocycling profile: an initial denaturation step at 94 °C for 3 min, followed by 35 cycles of denaturation at 94 °C for 1 min, annealing at 54 °C for 1 min, and extension at 72 °C for 1 min, with a final extension step at 72 °C for 7 min. PCR products were purified (Ampure XP Kit, Beckman Coulter, Indianapolis, USA) and sequenced in both directions on a 3730 l DNA Analyzer 96-capillary sequencer (Applied Biosystems, Foster City, CA, USA). We used CodonCode Aligner version 3.7.1 software (Codon Code Corporation, Dedham, MA, USA) to edit sequences and compared them to the GenBank database content using BLAST. The newly generated sequences are deposited in the GenBank database under the accession numbers OQ919798-OQ919804, OQ919806, OR245546, and OR245386.

Phylogenetic analyses were performed using the newly generated sequences of *D. ruber* and those for Derogenidae species available in GenBank (Table 1). Alignments for each gene region were constructed in AliView [28] and trimmed to the length of the shortest sequence. Nucleotide substitution models for phylogenetic analyses using the maximum-likelihood method were estimated using MEGA11 [29]. The best-fit models selected were the Kimura 2-parameter model with gamma distributed amongst-site rate variation (K2 + G) for the 28S rDNA alignment, Kimura 2-parameter (K2) model for the ITS2 alignment, and Tamura-Nei model (TN93) with estimates of invariant sites and gamma distributed amongst-site rate variation (HKY + I + G) for *cox*1. All trees were constructed in MEGA11, with 500 replications. Genetic distances [uncorrected p-distance model (Kimura 1980)] were computed with MEGA11.

**Table 1:** Hosts, locality, and GenBank accession data for the sequences of *Derogenes* spp. and halipegine derogenids analysed in this study

Species/lineage	Host	Locality	GenBank ID			Source
			28S rDNA	ITS2 rDNA	*cox*1	
*D. ruber*	*Chelidonichthys lastoviza*	Western Mediterranean, off Algeria	OQ919799	OQ919806	OR245386	Present study
	*Chelidonichthys lastoviza*	Western Mediterranean, off Algeria	–	OQ919798	OR245546	Present study
	*Chelidonichthys lastoviza*	Western Mediterranean, off Algeria		OQ919804		Present study
	*Chelidonichthys lastoviza*	Western Mediterranean, off Algeria	OQ919800	OQ919801	OQ919800	Present study
	*Chelidonichthys lastoviza*	Western Mediterranean, off Algeria	OQ919803	OQ919802		Present study
*D. varicus* lineage DV1	*Limanda limanda*	White Sea, Keret Archipelago	-		OM807173	[14]
	*Gadus morhua*	White Sea, Keret Archipelago	–	OM762003	–	[14]
	*Anarhichas lupus*	White Sea, Keret Archipelago	OM761965	OM762005	OM807176	[14]
	*Limanda limanda*	White Sea, Keret Archipelago	–	OM762006		[14]
	*Eleginus nawaga*	White Sea, Keret Archipelago	OM761967	OM762007	OM807178	[14]
	*Limanda limanda*	White Sea, Keret Archipelago	OM761968	–		[14]
	*Clupea pallasii*	White Sea, Keret Archipelago	OM761969	OM762009		[14]
	*Clupea pallasii*	White Sea, Keret Archipelago	–	–	OM807181	[14]
	*Gadus morhua*	Barents Sea, Dalniye Zelentsy	OM761971	–	OM807182	[14]
	*Myoxocephalus scorpius*	Barents Sea, Dalniye Zelentsy	OM761973	OM762013	OM807184	[14]
	*Triglops murrayi*	White Sea, Keret Archipelago	OM761976	OM762016		[14]
	*Gadus morhua*	White Sea, Velikaya Salma Strait	–	OM762015		[14]
	*Merlangius merlangus*	Skagerrak, North Sea	–	–	OQ916450	[15]
	*Merlangius merlangus*	Skagerrak, North Sea	–	–	OQ916440	[15]
	*Merlangius merlangus*	Skagerrak, North Sea	–	–	OQ916442	[15]
	*Merlangius merlangus*	Skagerrak, North Sea	–	–	OQ916445	[15]
	*Merlangius merlangus*	Skagerrak, North Sea	–	–	OQ916444	[15]
	*Merlangius merlangus*	Skagerrak, North Sea	–	–	OQ916437	[15]
*D. varicus* lineage DV2	*Hippoglossoides platessoides*	North Sea	AY222189			[45]
	*Buccinum scalariforme*	White Sea, Keret Archipelago	OM761977 ^a^	OM762017 ^a^		[14]
	*Amauropsis islandica*	White Sea, Keret Archipelago	OM761989	OM762029		[14]
	*Euspira pallida*	White Sea, Keret Archipelago	–	OM762030	OM807194	[14]
	*Euspira pallida*	Russia	–	OM762031	OM807195	[14]
*D. varicus* lineage DV3	*Eumicrotremus fedorovi*	North Pacific	MW504598	–		[46]
	*Eumicrotremus fedorovi*	North Pacific	MW504599	–		[45]
*D. lacustris*	*Oncorhynchus mykiss*	Argentina ^b^			LC586095	[31]
	*Salvelinus fontinalis*	Argentina ^b^			LC586094	[31]
	*Percichthys trucha*	Argentina ^b^			LC586093 LC586096	[31]
	*Galaxias maculatus*	Argentina ^b^	LC586089		LC586092	[31]
	*Galaxias maculatus*	Argentina ^b^	LC586090		LC586097	[31]
	*Galaxias maculatus*	Argentina ^b^			LC586098	[31]
*Allogenarchopsis problematica*	*Semisulcosipra reiniana*	East China Sea	MH628313			[46]
*Genarchopsis chubuensis*	*Rhinogobius flumineus*	East China Sea	MH628311			[46]
*Genarchella* sp. 1	*Herichthys labridens*	North-West Atlantic, off Yucatan	MK648276			[47]
*Genarchella* sp. 1	*Astyanax aeneus*	North-West Atlantic, off Yucatan	MK648277			[47]
*Thometrema lotzi*	*Lepomis microlophus*	North-West Atlantic, off Mississipi,	KC985236			[48]
*Thometrema patagonica*	*Percichthys trucha*	Argentina ^b^	LC586091			[31]
*Prosogonotrema bilabiatum* (outgroup)	*Caesio cuning*	Pacific Ocean	AY222191			[45]
*Accacladocoelium macrocotyle* (outgroup)	*Mola*	Western Mediterranean		KF687303		[49]
*Didymocystis wedli* (outgroup)	*Thunnus orientalis*	East China Sea			AB725624	Unpublished

^a^Two sequences by Krupenko *et al.* [14] are wrongly annotated on GenBank: OM761977.1 and OM762017.1, and these two *Derogenes varicus* complex sp. DV1 isolates are in fact DV2

^b^Rivers and lakes in Patagonia

## Results

**Family Derogenidae Nicoll, 1910**

**Subfamily Derogeninae Nicoll, 1910**

**Genus** ***Derogenes*** **Lühe, 1900**

**Fig. 1:**
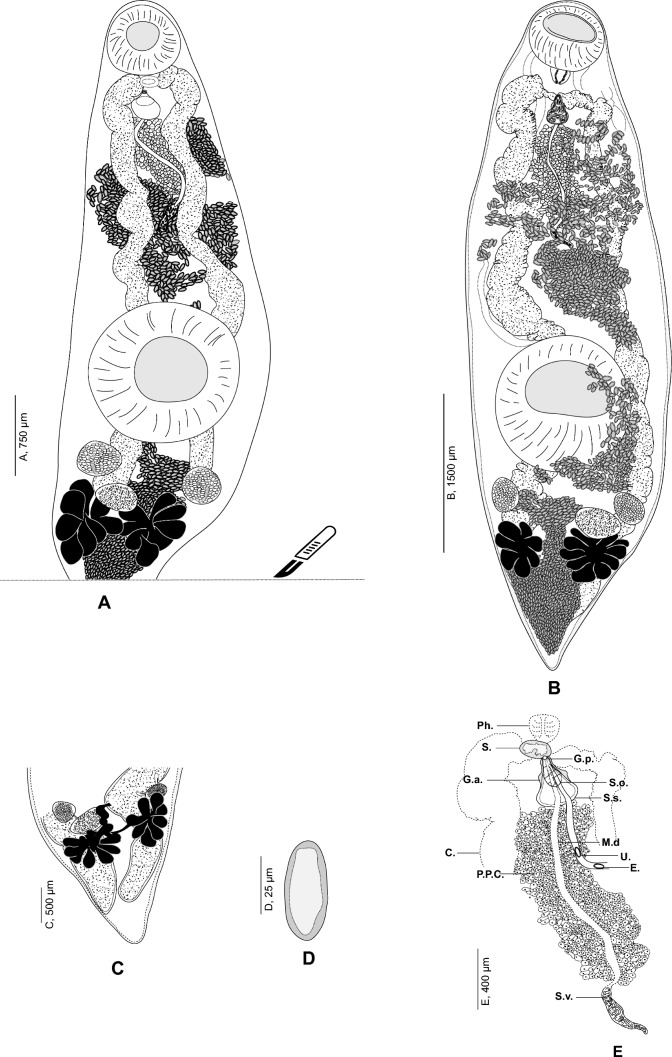
*Derogenes ruber* from *Chelidonichthys lastoviza*. **A** Hologenophore, ventral view, SMNH 218782. **B** Whole-body, ventral view, SMNH 218789. **C** Posterior extremity showing ends of caeca. **D** Egg, SMNH 218789. **E** Anterior extremity showing details of terminal genitalia, SMNH 218789. *Abbreviations*: C.: caecum; E.: egg; G.a.: genital atrium; G.p.: genital pore; M.d.: male duct; Ph.: pharynx; P.P.C.: prostatic cells; S.: sphincter; S.s.: sinus-sac; S.o.: sinus-organ; S.v.: seminal vesicle; U.: uterus

***Derogenes ruber* Lühe, 1900 (**Fig. 1** A-E)**

*Type-host*: Streaked gurnard *Chelidonichthys lastoviza* (syn*. Trigla lineata*) [16].

*Other reported host*: Piper gurnard *Trigla lyra* [17].

*Type-locality*: Off Rovinj, Croatia, Adriatic Sea [16].

*Other localities*: Off Split, Croatia, Adriatic Sea [17]; off Azores, Canary and Cape Verde islands [18] and off Spain [19], North-East Atlantic; Off Trieste, Italy, Western Mediterranean [15]; off Algeria, Western Mediterranean, present study.

*Site in host*: Gall bladder.

*Other sites in host*: Intestine [15].

*Voucher material*: A total of 25 voucher specimens are deposited in the collections of the Swedish Museum of Natural History, Stockholm (SMNH 218781- SMNH 218805) including 5 hologenophores (SMNH 218785, GenBank OR245386, OQ919806, OQ919799; SMNH 218786, GenBank OR245546, OQ919798; SMNH 218782, GenBank OQ919801, OQ919800; SMNH 218785, GenBank OQ919804; SMNH OQ919802, OQ919803).

### Redescription

[Based on 20 specimens mounted *in toto* and 5 hologenophores, metrical data are provided in Table 2.] Body stout, fusiform (Fig. 1A, B), widest at ventral sucker level. Tegument smooth. Pre-oral lobe present. Oral and ventral suckers well developed; oral sucker ventro-subterminal, subglobular-to-globular, wider than long; ventral sucker larger than oral sucker, spherical, located in posterior half of body. Forebody somewhat longer than hindbody. Prepharynx absent. Pharynx well developed, subglobular, muscular. Oesophagus short, barely visible, opening posteriorly via sphincter (Fig. 1E) to join intestinal bifurcation in anterior half of forebody, immediately posterior to pharynx. ‘Drüsenmagen’ not observed. Caeca broad, thick walled, extending into hindbody, reaching beyond gonads, terminating close to posterior extremity (Fig. 1C). Termination of caeca often obscured by eggs.

**Table 2:** Metrical data for *Derogenes ruber* from *Chelidonichthys lastoviza* and *Trigla lyra*

Host	*C. lastoviza*	*C. lastoviza*	*Trigla lyra*
Locality	Off Rovinj, Croatia	Off Split, Croatia	
No. of specimens	(*n* = 17)	(*n* = 2)	(*n* = 2)
Source	Present study	[16]	[17]
	Range (Mean)	Range	Range
Body	2679–5558 × 830–2040 (4348 × 1443)	5000–6000 × 2000	4200–4500 × 1300–1800
Forebody length	1240–2693 (1966)	–	–
Hindbody length	802–2133 (1364)	–	–
Pre-oral lobe length	15–71 (39)	–	–
Oral sucker	254–655 () × 364–741 (469 × 538)	600	505 × 505
Ventral sucker	546–1325 () × 547–1476 (952 × 1022)	750	950–1290
Pharynx	107–188 () × 86–221 (153 × 157)	200	168
Seminal vesicle	120–710 () × 38–170 (260 × 68)	–	–
Pars prostatica length	320–848 (565)	–	–
Right testis	150–395 () × 113–257 (233 × 170)	–	252 × 168
Left testis	145–396 () × 96–267 (247 × 170)	–	196 × 140
Ovary	143–459 () × 99–276 (293 × 183)	–	252 × 252
Right vitelline mass	251–843 () × 218–634 (432 × 412)	450	440 × 420
Left vitelline mass	254–620 () × 184–687 (452 × 424)	450	470 × 440
Individual vitelline lobe	122–422 () × 69–315 (247 × 146)	150–200	–
Eggs	49–60 × 26–38 (54 × 29) (*n* = 17)	56–36	23 × 23
Right testis to ventral sucker	53–298 (169)	–	–
Left testis to ventral sucker	13–239 (126)	–	–
Right post-testicular region length	646–1965 (1172)	–	–
Left post-testicular region length	730–1651 (1137)	–	–
Post-ovarian region length	523–1463 (897)	–	–
FO/BL (%)	34–53 (45)	–	–
RT/BL (%)	1–6 (4)	–	–
LT/BL (%)	1–5 (3)	–	–
RPT/BL (%)	17–36 (27)	–	–
LPT/BL (%)	19–31 (26)	–	–
OV/BL (%)	13–27 (20)	–	–
Sucker–length ratio	1:1.17–3.06 (1:2.00)	–	–
Sucker–width ratio	1:1.45–2.35 (1:2.00)	–	–

Abbreviations: *FO/BL (%)* forebody length as a percentage of body length, *RT/BL (%)* right testis length as a percentage of body length, *LT/BL (%)* left testis length as a percentage of body length, *RPT/BL (%)* right post-testicular region length as a percentage of body length, *LPT/BL (%)* left post-testicular region length as a percentage of body length, *OV/BL (%)* post-ovarian field length as a percentage of body length.

Testes two, entire, rounded, symmetrical, pre-ovarian, posterior to ventral sucker and separated by uterine coils. Seminal vesicle external, tubular, thin-walled, in forebody. Pars prostatica long, tubular, surrounded by numerous gland cells, extends between distal end of seminal vesicle and sinus-sac. Metraterm protruding along with ejaculatory duct into sinus-sac forming hermaphroditic duct (Fig. 1E). Sinus-sac muscular. Sinus-organ muscular, conical, projecting into genital atrium. Genital pore ventro-median, posterior to pharynx, at level of intestinal bifurcation (observed only in five specimens).

Ovary transversely-oval, sinistral, post-testicular, at 1110 from posterior extremity. Oviduct, oötype, and Laurer’s canal not observed. Uterus well developed, coiled throughout much of hindbody and in forebody as far as level of sinus-sac. Vitellarium comprises two symmetrical, subglobular, multi-lobed, post-ovarian masses; right vitelline mass composed of 8–10 lobes; left vitelline mass composed of 7–9 lobes. Eggs numerous, small, tick-shelled, without opercular spines or filaments (Fig. 1D).

Excretory vesicle Y-shaped; bifurcation not observed; arms unite dorsally to oral sucker in forebody (Fig. 1B); excretory pore terminal.

### Molecular Characterisation of the Digeneans

**Fig. 2:**
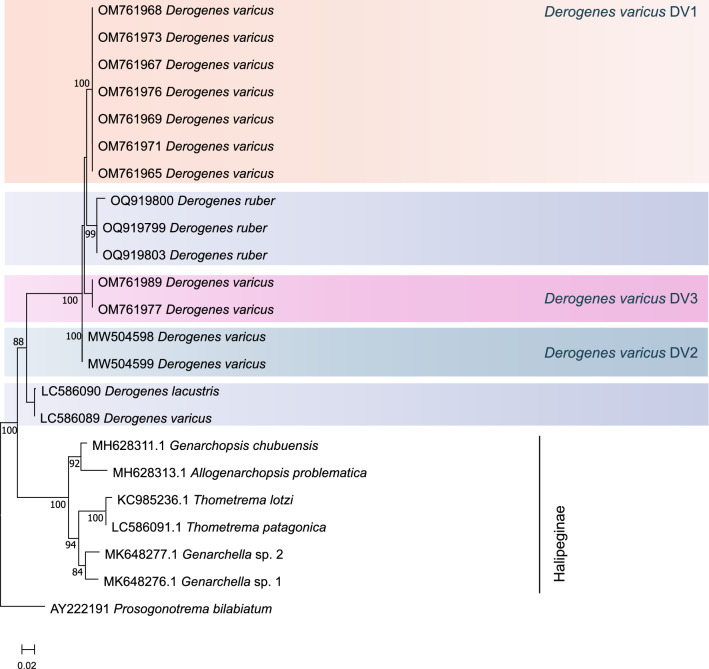
Tree inferred using the maximum-likelihood method based on the 28S rDNA sequence data; only bootstrap values higher than 70 are indicated. The newly generated sequences are indicated in red. Lineages “*Derogenes varicus* DV1, DV2, DV3” and *Derogenes lacustris* are highlighted in differently colored boxes

Four sequences (∼841 bp) for the nuclear 28S rRNA gene were obtained for *D. ruber*. The tree built using the newly generated sequences plus 20 sequences for species of *Derogenes* and the subfamily Halipeginae and *Prosogonotrema bilabiatum* Vigueras, 1940 as the outgroup yielded the topology shown in Fig. 2. There were a total of 688 positions in the final dataset. The general topology of the ML tree agreed with the taxonomic classification of the included species and distinct lineages. Species/lineages of *Derogenes* were recovered in five strongly supported reciprocally monophyletic clades: (i) *D. ruber* from *C. lastoviza* off Algeria; (ii) *D. lacustris* Tsuchida, Flores, Viozzi, Rauque et Urabe, 2021from *Galaxias maculatus* (Jenyns) off Argentina [31]; (iii) Lineage “*D. varicus* DV1” from fish hosts in the White and Barents seas [14]; (iv) Lineage “*D. varicus* DV2” from mollusc hosts in the White Sea [14]; and (v) Lineage “*D. varicus* DV3” from *Eumicrotremus fedorovi* Mandrytsa. in the Pacific Ocean [32]. All *Derogenes* spp. lineages (Derogeninae) clustered in a strongly supported clade distinct from that of the representatives of the Halipeginae.

The four newly generated 28S sequences of *D. ruber* were identical. They differed from Lineage “*D. varicus* DV1” from various fish hosts in the White and Barents seas (see above) by 2% (16 substitutions); from Lineage “*D. varicus* DV2” from *Hippoglossoides platessoides* (Fabricius) from North Sea and from a mollusc *Buccinum scalariforme* Møller. from the White Sea by 3% (20 substitutions); and from lineage “*D. varicus* DV3” by 2% (16 substitutions). Sequences of *D. ruber* differed from those of *D. lacustris* from *G. maculatus* (Jenyns) off Argentina by 9% (68 substitutions). Intraspecific/intralineage divergence for *Derogenes* spp./lineages ranged between 0 (for *D. varicus* lineages DV1, DV2, and DV3) and 1 substitution (for *D. ruber* and *D. lacustris*).

**Fig. 3:**
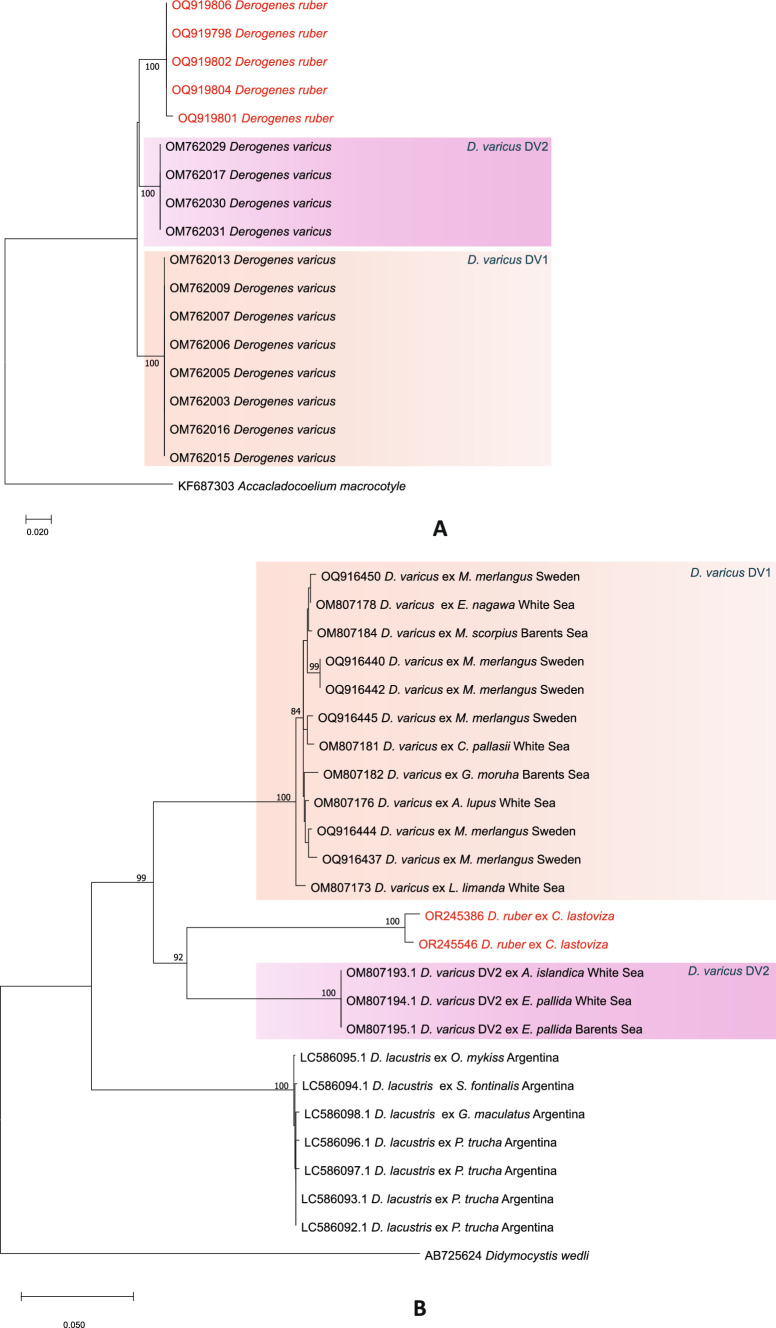
Trees inferred using the maximum-likelihood method based on the ITS2 rDNA and *cox*1 sequence data. A, ITS2 rDNA tree; only bootstrap values > 70 are indicated. The newly generated sequences are indicated in red. Lineages “*D. varicus* DV1 and “*D. varicus* DV2” are highlighted in differently colored boxes. There were no ITS2 sequences available for *D. lacustris*. B, *cox*1 tree; only bootstrap values > 70 are indicated. The newly generated sequences are indicated in red. *Derogenes lacustris* and lineages “*D. varicus* DV1”, “*D. varicus* DV2” are in different colors. There were no *cox*1 sequences available for the lineage “*D. varicus* DV3"

Five ITS2 sequences (∼566 bp) were obtained for *D. ruber*. The tree built using the newly generated sequences aligned with 12 sequences for *Derogenes* spp. and *Prosogonotrema bilabiatum* as the outgroup is shown in Fig. 3A*. Derogenes ruber* and the lineages “*D. varicus* DV1” from various fish hosts in the White and Barents seas and “*D. varicus* DV2” from the molluscs *B. scalariforme*, *Amauropsis islandica* (Gmelin) and *Euspira pallida* (Broderip & Sowerby) from the White and Barents seas clustered in reciprocally monophyletic groups with a maximum nodal support.

The five newly generated ITS2 sequences for *D. ruber* were also identical and differed from those for the lineage “*D. varicus* DV2” by 4% (16 substitutions) and from those for the lineage “*D. varicus* DV1” by 5% (21 substitutions). None of the taxa included in the analysis showed intraspecific/intralineage variation.

The two newly generated *cox*1 sequences of *D. ruber* (∼898 bp) were identical. We also included in the analysis four sequences of *D. varicus* (sensu stricto) from *Merlangius merlangus* (L.) from off Sweden [15]. The tree built using the newly generated sequences aligned with 22 sequences for *Derogenes* spp. and *Didymocystis wedli* Ariola, 1902 as the outgroup is shown in Fig. 3B. The species/lineages of *Derogenes* formed four reciprocally monophyletic groups with maximum support: (i) *Derogenes lacustris* from salmonids off Argentina; (ii) *D. ruber* from *C. lastoviza* off Algeria; (iii) Lineage “*D. varicus* DV1” from *M. merlangus* off Sweden, and fish hosts in the White and Barents seas [14]; and (iv) Lineage “*D. varicus* DV2” from mollusc hosts in the White Sea [32]. The intraspecific divergence between the newly generated *cox*1 sequences for *D. ruber* was 0.8% (7 substitutions). Sequences of *D. ruber* differed from the sequences for lineages “*D. varicus* DV1” and “*D. varicus* DV2” by 19% (158 substitutions) and 17% (135 substitutions), respectively. The largest genetic divergence was found between *D*. *ruber* and *D. lacustris* (23%; 186 substitutions). Intraspecific/intralineage divergence for *Derogenes* spp./lineages ranged between 0% (Lineage “*D. varicus* DV2”) and 2% (*D. ruber*: 0.8%; *D. lacustris*: 0.1–0.2; Lineage “*D. varicus* DV1”: 1%).

## Discussion

*Derogenes ruber* was described from the gall bladder of the streaked gurnard *C. lastoviza* off Rovinj, Croatia, Adriatic Sea [16]. Although the original description of *D. ruber* was detailed, it lacked illustrations. The only subsequent illustration of this species is that of Sey [17], which barely shows any internal organs and omits any details of the terminal genitalia. Sey (1968) examined three specimens of a distinct host, *T. lyra*, and redescribed briefly *D. ruber* based on two specimens. Although the geographical distribution of the type-host, *C. lastoviza*, is wide, *D. ruber* has been reported only from the Central Mediterranean (Adriatic Sea off Croatia, type-locality in the original description [16] and later, from a different host [17] and recently from the type-host off Italy, based on A. Looss’s material) [15]. The latest record despite providing few morphometrical data and illustration did not include any genetic data. *Derogenes ruber* was reported from the type-host in the North-East Atlantic, off Azores, Canary and Cape Verde islands [18] and off Spain [19]. Consequently, this paper provides a detailed illustrated description of *D. ruber* and Algeria as a new locality for this digenean. Additionally, we genetically characterised for the first time *D. ruber* using the partial fragments of the nuclear 28S rRNA gene and ITS2, and the mitochondrial *cox*1 gene. Most sequences for *Derogenes* spp. available to date are those provided in an extensive study by Krupenko *et al.* [14] and Tsuchida *et al.* [31] who provided abundant data, corresponding to the “candidade” *D. varicus* species complex and *D. lacustris*, respectively. Krupenko *et al.* [14] have shown the existence of four groups (labelled as DV1-DV4) within the “candidade” *D. varicus* species complex; of these, they considered that two (DV1 and DV2) may belong to distinct species [14]. Recently, Bouguerche *et al.* [15] demonstrated that DV1 is in fact *D. varicus *sensu stricto.

Herein, the 28S rDNA analysis recovered *D. ruber* in a clade distinct from lineages “*D. varicus* DV1, DV2, and DV3” and the well-established species *D. lacustris*. The ITS2 analysis supported the monophyly of *D. ruber*, and lineages “*D. varicus* DV1” and “*D. varicus* DV2” and the *cox*1 tree yielded a similar topology. Although the sequences obtained herein were short affecting thus the alignment’s length, the analysis led to results similar to those of Krupenko *et al.* [14].

More importantly, the genetic distance for the *cox*1 gene between *D. ruber* and lineages “*D. varicus* DV1” and “*D. varicus* DV2” was 19% and 17%, respectively; *D. ruber* also differed from *D. lacustris* by 23%. These levels of genetic divergence agree well with previously reported interspecific divergence based on *cox*1 within the closely related halipegine derogenids ranging between 10.5–15.1% for *Genarchopsis* spp. [23] and 16.9–20.4% for *Genarchopsis* Ozaki, 1925 and *Allogenarchopsis* Urabe & Shimazu, 2013 [33]. Furthermore, the levels of interspecific genetic divergence are more than ten times greater than those for the intraspecific divergence for the mitochondrial “barcode” marker, thus supporting the recognition of *D. ruber* as a valid distinct species. The molecular data generated herein advance our knowledge on interspecific genetic variations within *Derogenes* and will help further efforts to untangle the *D. varicus* species complex and delimit the potentially cryptic species hidden under the single name “*D. varicus*”. Additionally, the morphometrical data of *D. ruber* from the type-host (Table 2) will help accessing interspecific morphological differences.

A problem arises when comparing *D. ruber* to a closely related species, *D. latus* Janiszewska, 1953, first described based on a single specimen in the intestine of *Mullus barbatus* Linnaeus from the same Adriatic locality as that of *D. ruber*, off Split, Croatia [34]. *Derogenes latus* was redescribed from the intestine of *M. barbatus* and *Trisopterus capelanus* (Lacépède) in the North Adriatic Sea [35] and from the gall bladder of *M. surmuletus* off Corsica (France), Western Mediterranean [10]. The redescription provided by Bartoli and Gibson [10] (based on accessible voucher material and serial sections) should undoubtfully be referred to as the most detailed modern redescription of *D. latus*. *Derogenes latus* has been frequently reported from its type-host in the Western Mediterranean, off Spain [36] and off France [37], and from a closely related host, *M. surmuletus*, in the Western Mediterranean (off France and Algeria) [37–39].

This species has also been reported on hosts other than Mullidae, mainly from S. *scrofa* (Scorpaenidae) in the Western Mediterranean, off Spain [40] and off France [41]; from *L. mormyrus* (Sparidae) off Montenegro, Adriatic Sea [42] and off Algeria, Western Mediterranean [43]. It was furthermore recorded from *Sardinella aurita* Valenciennes. (Dorosomatidae) off Algeria, Western Mediterranean [44] and from *Phycis phycis* (Linnaeus) (Phycidae) from the Western Mediterranean (off France) [41].

The taxonomic status of *D. latus* is uncertain. The distinction *D. ruber* and *D. latus* has been questioned [10], and the two species share a stout body, post-testicular vitellarium composed of two multi-lobed masses and a uterus occupying almost the entire body [10, 35, 42]. The type-hosts are, however, different: *C. lastoviza* for *D. ruber* [16] and *M. barbatus* for *D. latus* [34]. Overall, all morphometric data for *D. ruber* and *D. latus* overlapped **(**Tables 2, 3**)** except for specimens of *D. latus* from *M. surmuletu*s and *S. scrofa* from the Western Mediterranean having larger eggs (see Table 3) and the two species clearly share the deeply loped shape of the vitelline masses. It is worth noting that a comparison of the present specimens of *D. ruber* with those of *D. latus* provided by Bartoli and Gibson [10] in the most detailed modern description based on accessible voucher material and serial sections and providing metrical data, revealed that, despite some overlaps, *D. latus* is generally larger than *D. ruber* (means 5581 × 2180 *vs*. 4348 × 1443 µm) with a longer forebody (mean 2523 *vs*. 1966 µm) and longer hindbody (mean 1926 *vs*. 1364 µm). *Derogenes latus* also differs from *D. ruber* in having a broadly longer pre-oral lobe (mean 126 *vs*. 39 µm), larger oral sucker (means 787 × 789 *vs*. 469 × 538 µm), larger ventral sucker (means 1136 × 1110 *vs*. 952 × 1022 µm), and larger pharynx (means 291 × 254 *vs*. 153 × 157 µm). Additionally, *D. latus* differs from *D. ruber* in having a longer pars prostatica (mean 790 *vs*. 565 µm), considerably larger testes (means 480 × 366 *vs*. 233 × 170 µm for right testis, 488 × 400 *vs*. 247 × 170 µm for left testis), larger ovary (means 511 × 341 *vs*. 293 × 183 µm), and larger vitelline masses (means 823 × 500 *vs*. 432 × 412 µm for right vitelline mass, 963 × 608 *vs*. 452 × 424 µm for left vitelline mass).

**Table 3:** Metrical data for *Derogenes latus* from different hosts and localities in the Mediterranean

Host	*Mullus barbatus*	*Mullus barbatus, Trisopterus capelanus*	*Mullus surmuletus*	*Scorpaena scrofa*	*Lithognathus mormyrus*
Habitat	Intestine	Intestine	Gall-bladder	Gall-bladder	Stomach
Locality	Off Split, Croatia, Adriatic Sea	North Adriatic Sea	Off Corsica, France, Western Mediterranean	Off Spain, Western Mediterranean	Off Montenegro, Adriatic Sea
No. of specimens	(*n* = 1)	na	(*n* = 5)	(*n* = 12)	(*n* = 1)
Source	[34]	[35]	[10]	[40]	[42]
			Range (Mean)	Range (Mean)	
Body			4165–7120 × 1700–2850 (5581 × 2180)	5414–5983 × 1845–1851 (5698 × 1848)	9300 × 2550
Forebody			2000–3295 (2523)		
Hindbody			1320–2510 (1926)		
Pre-oral lobe			110–150 (126)		
Oral sucker	500 × 450	500 × 450	660–980 × 712–893 (787 × 789)	703–726 × 750–832 (714 × 791)	600 × 620
Ventral sucker	800 × 630	800 × 630	850–1340 × 915–1275 (1136 × 1110)	1336–1371 × 1429–1476 (1353 × 1453)	900 × 920
Pharynx	180 × 160	180 × 630	240–362 × 213–293 (291 × 254)	210–216 × 193–240 (213 × 216)	270 × 300
Seminal vesicle			350–560 × 110–170 (445 × 130)	293–369 (331)	
Pars prostatica			533–1013 (790)	468–556 (512)	
Right testis	340 × 190	340 × 190	350–640 × 270–430 (480 × 366)	210–234 (222)^a^	400 × 370
Left testis			373 × 680 × 320–510 (488 × 400)	205–304 (254)^a^	
Sinus-organ	170 × 200	170 × 200	150–240 × 150–270 (215 × 210)		310 × 120
Ovary	340 × 110	340 × 100	426–560 × 240–453 (511 × 341)	246–310 (278)^a^	450 × 500
Right vitelline mass	540 × 350	540 × 350	600–1170 × 320–700 (823 × 500)		
Left vitelline mass			550–1550 × 530–850 (963 × 608)		
Eggs	50 × 26	50 × 25	59–74 × 33–43 (68.5 × 39.5)	64–70 (67) × 35	50–53 × 25–26
Oral sucker to genital pore			80–373 (180)		

Bartoli and Gibson [10] convincingly highlighted the striking morphological similarity between *D. latus* and *D. ruber* and indicated that the egg size given by Sey [17] for *D. ruber* is probably an inaccuracy. They refrained from synonymising the two species formally until further studies of material from the type-hosts and localities are available. Although we found morphometric differences between the present material of *D. ruber* from the type-host and the material of *D. latus* described by Bartoli and Gibson [10], and given that the occurrence of a single *Derogenes* species in various hosts has been challenged by molecular data [14, 15, 31], and both *D. lacustris* and *D. varicus *sensu stricto (*D. varicus* lineage DV1 of Krupenko *et al.* [14]) had been genetically proven to occur in various hosts (see Fig. 3A), it is possible that *D. ruber* and *D. latus* are indeed synonymous, thus transforming *D. ruber* to a euryxenic species. However, since molecular data for *D. latus* are still lacking, we also refrained from synonymising the two species. The genetic data generated herein for D*. ruber* from its type-host will be certainly valuable for a future investigation of the synonymy of these two species.

## Data Availability

All relevant data are within the paper.
